# Eco-Friendly Fungal Chitosan-Silica Dual-Shell Microcapsules with Tailored Mechanical and Barrier Properties for Potential Consumer Product Applications

**DOI:** 10.1021/acsomega.4c02287

**Published:** 2024-06-20

**Authors:** Daniele Baiocco, Mohammed Al-Sharabi, Benjamin T. Lobel, Olivier J. Cayre, Alexander F. Routh, Zhibing Zhang

**Affiliations:** † School of Chemical Engineering, University of Birmingham, Birmingham B15 2TT, U.K.; ‡ Department of Chemical Engineering and Biotechnology, University of Cambridge, Cambridge CB3 0AS, U.K.; § School of Chemical and Process Engineering, University of Leeds, Leeds LS2 9JT, U.K.

## Abstract

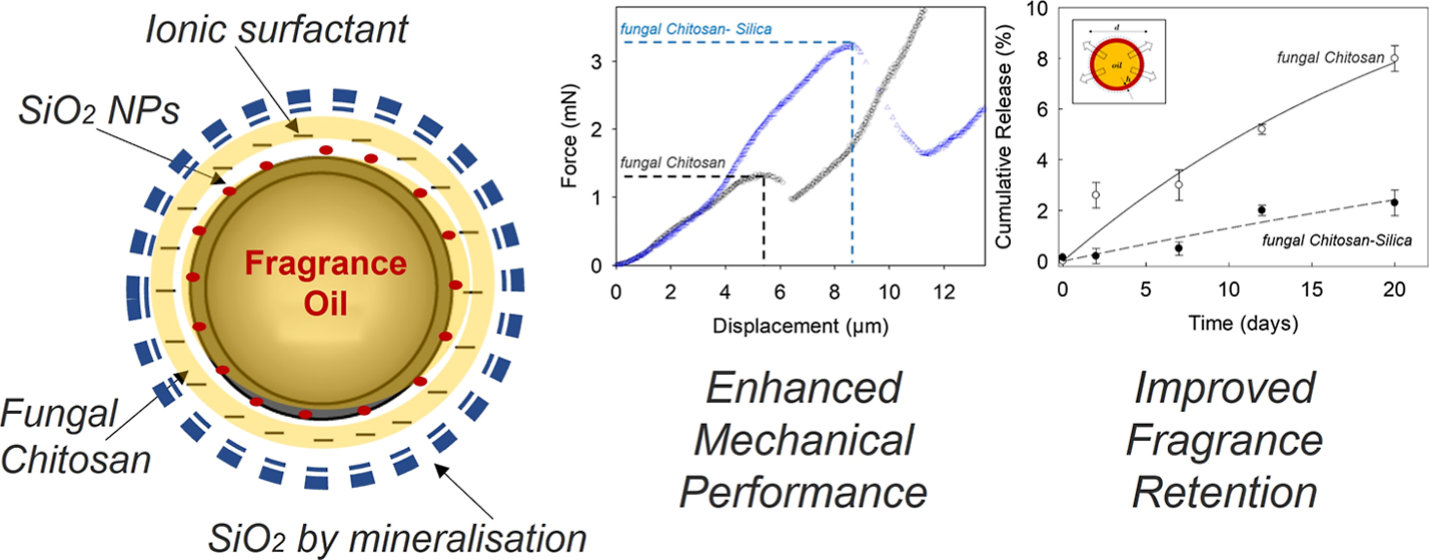

Commercial perfume microcapsules are becoming popular across the globe to fulfill consumers’ demands. However, most of microcapsules rely on synthetic polymers and/or animal-sourced ingredients to form the shells. Therefore, replacement of the shell materials is imperative to minimize environmental microplastic pollution, as well as to meeting peoples’ needs, religious beliefs, and lifestyles. Herein, we report a methodology to fabricate environmentally benign dual-shell (fungal chitosan-SiO_2_) microcapsules laden with fragrance oil (hexyl salicylate). Anionically stabilized oil droplets were coated with fungal chitosan via interfacial electrostatic interactions at pH 2, which were then covered by an inorganic coating of SiO_2_ produced via external alkaline mineralization of sodium silicate. Core–shell microcapsules with a spherical morphology were achieved. Under compression, dual-shell chitosan-SiO_2_ microcapsules yielded a mean nominal rupture stress of 3.0 ± 0.2 MPa, which was significantly higher than that of single-shell microcapsules (1.7 ± 0.2 MPa). After 20 days in neutral pH water, only ∼2.5% of the oil was released from dual-shell microcapsules, while single-shell microcapsules cumulatively released more than 10%. These findings showed that the additional SiO_2_ coating significantly enhanced both mechanical and barrier properties of microcapsules, which may be appealing for multiple commercial applications, including cosmetics and detergents.

## 1 Introduction

Fast-moving consumer goods (FMCG) are a well-established industry. Recently, there has been a critical transformation in consumer preferences, where the demand extends beyond basic functionality of the final product.^1^ To this end, a plethora of products in the cosmeceutical, personal care, household, and cleaning sectors are now designed with microencapsulation technologies to offer enhanced functionalities and extended shelf life of the final goods, reflecting a rapid evolution in the FMCG landscape.^2^ Synthetic polymers, such as polymethacrylates,^3^ polyurethanes,^4^ and melamine,^5^ have long dominated the field of materials science, engineering, and microencapsulated substances due to their versatility, ease of processing, mechanical robustness, thermo-chemical stability, and cost-effectiveness.^6^

In general, manmade resins can be tailored more easily to possess desirable physicochemical properties than natural materials, making them highly sought-after for various industrial applications.^7^ However, leave-on/rinse-off microparticles contained in many products, such as cosmetics, cleaning, and laundry formulations, may be classified as primary nonbiodegradable microplastics and, therefore, pose a severe threat to ecosystems when they enter wastewaters.^8^ In pursuit of sustainability, both European Union (EU) and non-EU countries have proactively committed to progressively implementing regulatory bans against microplastics in many personal care products.^9^

In light of this restriction, there is an urgent need to develop novel formulations based on environmentally benign materials to tackle this global issue, without undermining the performance of the final products. This includes for example high-performance green alternatives for topical fragrance delivery and skin exfoliation.^10^

Chitosan has shown promise for replacing synthetic polymers in certain cleaning and personal care formulations, like skin, hair, and cosmeceutical ointments.^11^ However, chitosan is traditionally sourced from the exoskeletons of crustaceans (e.g., crab, shrimp, and lobster) and has therefore faced limitations in cosmetic and nutraceutical applications due to potential zoonosis and ethical concerns associated with biodiversity and endangered species protection.^12^ Consequently, there has been a growing interest in using chitosan derived from plants or produced through biotechnological processes, for pharma-nutraceutical and cosmetic purposes. Notably, chitosan obtained via fungal fermentation has emerged as a promising candidate for cosmetic and personal care goods, with properties suitable for the production of mascaras, hair conditioners/foams, skin protecting lotions, and body creams.^13^

Fungal chitosan (fCh) is an animal-free, biocompatible, hypoallergenic, nontoxic, non-GMO, and fully biodegradable biopolymer. Recently, it has proven suitable for the microencapsulation of fragrance oils, with a potential for healthcare, biomedical, and cosmetic applications.^1,13^ However, as with many other (bio)polymers, it often requires the presence of petroleum-derived cross-linking agents (i.e., formaldehyde, glutaraldehyde) to be effective. This modifies the structure of the biopolymer significantly, possibly making it recalcitrant to degradation.^10^

Although biological nontoxic cross-linkers, such as transglutaminase, have also been employed, the performance properties of the resulting microcapsules are to date limited when compared to those of traditional microcapsules cross-linked using petrochemically derived agents.^14,15^ Thus, microencapsulation research is shifting toward more sustainable microcapsule designs, placing particular emphasis on hybrid/composite shells. Long et al.^16^ outlined a strategy to fabricate core–shell composite microcapsules. This approach entailed a calcium shellac matrix on top of primary CaCO_3_ nanoparticle (NPs)-stabilized microcapsules, resulting in reduced leakage of the hydrophobic core and high mechanical stability.

Another safe inorganic shell material extensively used for microcapsules is silica due to its benign environmental, abrasive, and hygroscopic properties. Moreover, it boasts remarkable thermal, chemical, and mechanical stability rendering it a promising candidate for applications in healthcare and cosmetics.^17,18^ Although the encapsulation of hydrophobic ingredients within silica shells has arisen in scientific literature, the intrinsic mesoporosity of silica can undermine the quality of the capsules, leading to comparatively high leakage rates.^17^ Clearly, this is undesirable in fragrance delivery, personal care, and cosmetic products, especially those designed for a shelf life of approximately 2–3 years.^19^ Hence, silica shells alone may not suffice in retaining active ingredients over a prolonged period. Similar conclusions have been drawn in the presence of alternative inorganic shell materials, such as calcium carbonate and calcium phosphate.^20,21^ Pickering emulsion (bio)polymerization (PEP) offers a green, versatile, and potentially scalable approach of synthesizing hybrid core–shell microcapsules, where a core (e.g., droplet) is armored by inorganic nanoparticulates, such as silica NPs. Unlike conventional organic emulsifiers, NPs adsorb onto the droplets, forming a robust protective layer, conducive to the mechanical strength of the resulting microcapsules.^22^ Pickering-stabilized droplets are often polymerized, using synthetic^23^ or preferably natural monomers^24^ to achieve architectures with improved surface functionalities, wettability, and tunable hydrophilicity/hydrophobicity. For example, Kanomata et al.^24^ reported the biomimetic polymerization of coniferyl alcohol into lignin around oil droplets stabilized by nanocellulose NPs. This green approach contributes to mitigating the risks associated with the release of common ionic/nonionic organic emulsifiers into the environment, which are typically used alone at high concentrations.^25^

Lately, the inter-relationship between silica and animal-sourced chitosan, and then the stability of the ensuing system, has been investigated by Matusiak et al.^26^ The authors reported that the addition of the cationic chitosan may play a crucial role in the structural and chemical stabilization of colloidal silica suspensions, with a potential for industrial applications, such as functionalized materials and catalysis. This was fulfilled via electrosteric functionalization of silica particles following the adsorption of chitosan chains, allowing for the modulation of the repulsive forces between the particles, and hence controlling their separation. Although promising, to the best of the authors’ knowledge, this system has not been applied to other research areas, such as fragrance encapsulation.

In this work, we propose a facile method to fabricate fungal chitosan-silica microcapsules featuring a core of hexyl salicylate (HS) as a model fragrance oil. Specifically, emulsified HS droplets were stabilized ionically and sterically using sodium dodecyl sulfate (SDS) and ε-polylysine-modified SiO_2_ NPs, respectively. Fungal chitosan was used to form an organic coating (primary shell) around the oil droplets at pH 2. The primary shell was then enveloped by an inorganic coating made of SiO_2_ (second shell) via the external alkaline (pH 11) mineralization of sodium silicate. The microcapsules were assayed for their morphological, barrier, and mechanical properties by bright field optical/fluorescence-sensing microscopy, scanning electron microscopy, UV–vis spectrophotometry, and a micromanipulation technique developed at the University of Birmingham, UK.^27^

## 2 Materials and Methods

### 2.1 Materials

Food-grade fungal chitosan (fCh; KiOsmetine-Cs, molecular weight <190 kDa, degree of deacetylation 80%; CAS no. 9012-76-4) was provided by Kitozyme S.A. (Herstal, Belgium, EU). Aerosil 300 (hydrophilic fumed silica NPs (SiO_2_ NPs) with an average particle diameter of <10 nm and specific surface area of ∼300 m^2^/g; CAS no. 7631-86-9) were obtained from Evonik Industries AG (Essen, Germany, EU). ε-Polylysine (εPLL) hydrochloride (≥95% on dry basis w/w; CAS no. 25988-63-0) was purchased from BOC Science (New York, NY, US). All other analytical grade reagents, including HS (>99.0%, density = 1040 kg m^–3^; CAS no. 6259-76-3), sodium silicate (Na_2_SiO_3_; CAS no. 1344-09-8), SDS (CAS no. 151-21-3), Nile Red (NR; CAS no. 7385-67-3), octan-1-ol (∼99%; CAS no. 111-87-5), fuming hydrochloric acid (36% w/v HCl; CAS no. 7647-01-0), sodium hydroxide (NaOH; CAS no. 1310-73-2), and sodium chloride (NaCl; CAS no. 7647-14-5) were purchased from Merck Ltd. & Sigma-Aldrich (Dorset, UK), stored according to the Safety Data Sheet guidelines, and used without further purification. All the solutions were formulated using ultrapure deionized water (CAS no. 7732-18-5; 18.2 MΩ cm^–1^ at 25 °C).

### 2.2 Preparation of Dual-Shell Microcapsules

**Figure 1:**
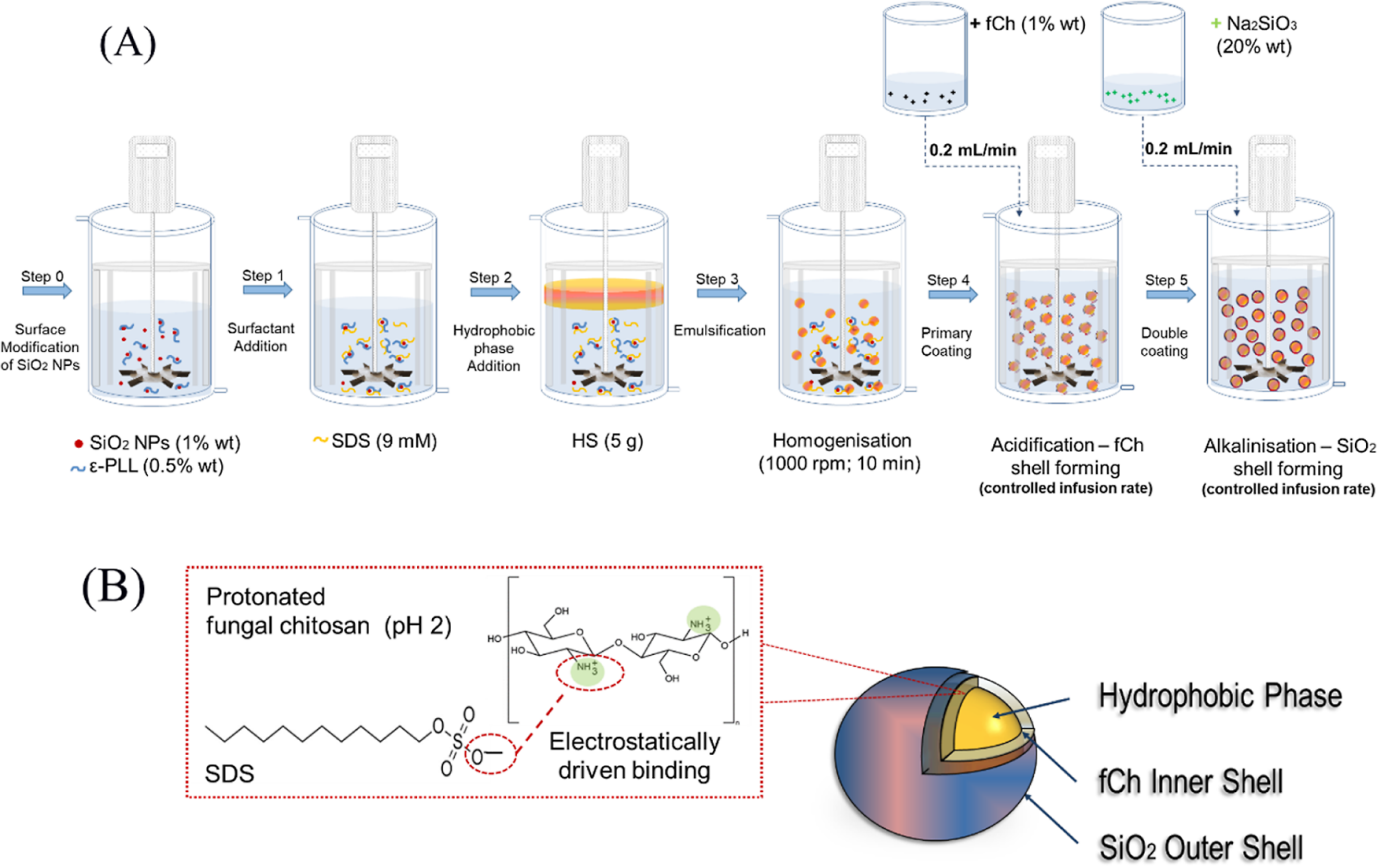
(A) Schematic diagram of the two-pot encapsulation of HS via emulsification (step 3) followed by electrostatically driven deposition of fCh as a primary coating (step 4) and subsequent external mineralization of SiO_2_ as a secondary coating using Na_2_SiO_3_ (step 5) and (B) schematic of the resulting dual-shell microcapsule with a hydrophobic core enwrapped by a primary fCh coating (inner shell) which is covered with a secondary coating of silica (outer shell); the zoomed-in inset of (B) highlights the possible mechanism of interaction between SDS and fCh.

An aqueous suspension of pristine SiO_2_ NPs (1.3 g) was generated under continuous stirring (600 rpm; Rushton turbine *Ø* 34 mm; IKA Eurostar 20, Germany, EU) over 10 min to efficient dispersion of the NPs in water (0.1 L) within a double-glazed cylindrical vessel (liquid height/tank diameter ∼1; clearance/impeller diameter ∼3/4) with four baffles (baffle width/Tank diameter ∼0.1). The suspension was then titrated to pH 7 using HCl_aq_/NaOH_aq_. Cationic εPLL (0.54 g) was readily dissolved in the aqueous medium and left to stir for an additional 10 min in order to allow the surface modification of the SiO_2_ NPs at pH 7. Subsequently, anionic SDS (0.25 g) at its critical micelle concentration (CMC ∼ 9 mM at 25 °C^28^) was added under stirring. The core oil (HS; 5 g) dyed with fluorescence sensing Nile Red (<5 mg; ∼0.1% *w*_NR_/*w*_HS_) was added to the suspension to generate oil-in-water (o/w) droplets under mechanical stirring (*Ø* 34 mm; 1000 rpm; 10 min). The size distribution of the droplets was measured by laser diffraction (Malvern Mastersizer 2000, Malvern Instruments, Malvern, England, UK). An acidic (pH 2) solution (20 mL) of fCh (1% w/w) was prepared using HCl_aq_ in a separate vessel. This was then added to the SDS–εPLL–SiO_2_NP HS emulsion at a controlled flow rate (200 μL min^–1^) through a preloaded disposable syringe using an infusion pump (Ultra 70-3007, Harvard Apparatus Inc., Holliston, MA, USA) under stirring (625 rpm) until completion (80 min). Consequently, the pH of the medium dropped to ∼2.5 affording fCh with an extended conformation to interact with SDS. This resulted in a primary coating formed around the oil droplets. Subsequently, a sodium silicate solution (20% w/w; 20 g) was gradually added (200 μL min^–1^) to trigger its acidic hydrolysis (Na_2_SiO_3_ + 2 HCl → SiO_2_ + 2 NaCl + H_2_O) under continuous stirring (400 rpm), allowing the precipitation of mineralized silica to form the secondary coating on the surface of the emulsion droplets. Schematics of the whole process and the ensuing dual-shell microcapsules are displayed in Figure 1A,B, respectively. The zoomed-in inset of Figure 1B shows the proposed mechanism of electrostatic interaction between fCh and SDS. All preparation was performed at 25 ± 0.1 °C (Dyneo DD-300F, Julabo Ltd., Stamford, UK).

### 2.3 Characterization: Analytical Techniques

#### 2.3.1 Optical and Fluorescence Microscopy

The morphology of single- and dual-shell microcapsules were assessed by bright-field microscopy (Leica DM-RBE, Leica Microsystems GmbH, Germany, EU) using the appropriate magnification lenses (PL-Fluotar, 5×/0.12, 10×/0.30, Leica, Germany, EU). Real-time digitalized image capturing of the microphotographs was enabled by a high-definition top-view camera (Moticam Pro252, Leica Microsystems Imaging Solution Ltd., UK) connected to its image analysis software (Motic Images Advanced 3.2, Motic China Group Ltd., Xiamen, China). The micrographs of fluorescence sensing microcapsules were allowed via a blue illumination beam (wavelength of 460 nm) operated by a light control pod (Cool LED pE-300^white^, Cool LED Ltd., Andover, UK). Additionally, the mean size of the droplets was analyzed via image analysis freeware (ImageJ 1.53c, National Institute of Health, Bethesda, MD, USA).

#### 2.3.2 Scanning Electron Microscopy

Scanning electron microscopy (SEM, TM3030Plus, Hitachi High Tech, Tokyo, Japan) integrated with an energy-dispersive X-ray (EDX) system operating at a working distance of ∼9 mm under an accelerating voltage of 15–30 kV was employed to investigate the morphological, structural, and surface topographical features of the microcapsules. The specimen was prepared by placing an aliquot (∼100 μL) of suspended microcapsules on to adhesive carbon-coated tab (Leit 9 mm disc, Agar Scientific, UK) attached to the metal stub. The sample was allowed to completely air-dry. Subsequently, the ensuing dry microcapsules were gold-coated via sputter deposition (Polaron Sputter Coater SC7640, QuorumTech, Sussex, UK) within a high vacuum (<10^–3^ Pa) chamber under moderate emission current (∼25 mA). This produced a thin conductive film (∼8 nm) to minimize any undesirable charging effects while imaging.

#### 2.3.3 Particle Size Analysis

The particle size and size distribution of microcapsules was investigated by laser diffraction using a continuously stirred (2000 rpm) sample dispersing unit (Hydro2000SM, Malvern Instruments Ltd., UK). Suspended microcapsules (∼5 mL) were loaded into the dispersing unit prefilled with ∼120 mL of ultrapure deionized water and then assayed for their particle size distribution. The refractive indices of fCh and SiO_2_ were 1.521 and 1.461, respectively.^10,29^ The Sauter (surface area weighted) mean diameter was evaluated. The measurements were carried out in triplicate.

#### 2.3.4 Net Electrokinetic Charge

Zeta potentiometry (ZP) is an electrophoretically mediated technique used to characterize the surface properties of (bio)polymers and other colloids by a virtual index, namely, the net electrokinetic charge (NEC) or zeta potential. ZP (Zeta Sizer Ultra, Malvern Panalytical, Malvern, UK) was used to measure the electrokinetic and/or zeta potential of εPLL solutions and SiO_2_ NP suspensions at 25 °C over the required pH range (2–12). Aqueous HCl/NaOH (0.1 M) were used to adjust the pH to the desired values. Ultrapure deionized water (18.2 MΩ cm^–1^) and NaCl (1 mM) as the background polyelectrolyte were used. Specially designed folded capillary measurement cells (DTS1070, Malvern, UK) were employed. They were thoroughly rinsed three times with ultrapure deionized water prior to inoculating the specimen.

#### 2.3.5 Encapsulation Efficiency and Payload

The encapsulation efficiency (EE) and payload of microcapsules was evaluated by UV–vis according to our previous studies.^10^ Briefly, an aliquot (50 mL) of fCh-SiO_2_ microcapsule suspension was filtered (mesh size ∼2 μm, Whatman grade 6, Cytiva, UK) by suction (maximum pressure 50 kPa, N938 Laboport, KNF, Freiburg, Germany, EU). A microcapsule slurry was collected and washed with ultrapure water. Subsequently, 0.25 g of microcapsule slurry was loaded into a screw capped bottle and then dispersed into 36% (w/w) aqueous propanol (50 mL) as a receptor medium. The liquid medium was contacted to an ultrasonic processor probe (130 W, 20 kHz; amplitude 60%; 2:1 (s) pulse sequence; probe diameter 20 mm; Vibracell, Sonics & Materials, Inc., Newtown, CT, US) at ambient temperature to allow the rupture of the shells. The resulting shear forces and local microstreaming allowed for releasing the active material (HS) from the disrupted microcapsules into aqueous propanol. Following the ultrasonication, damaged microcapsule shells were separated out by centrifugation (refrigerated benchtop centrifuge, SIGMA, 2-16 KL, Germany, EU) to afford a clear HS-solubilized supernatant. This was analyzed by UV–vis spectroscopy. The absorbance of each supernatant was recorded at 306 nm (absorbance peak of HS) using a spectrophotometer (CE 2021, Cecil Instruments Ltd., UK). A standard calibration curve (coefficient of determination *R*^2^ ≥ 0.98), based on known concentrations of HS with their corresponding absorbance values, was constructed to estimate the concentration of HS in aqueous propanol (36% v/v). The experiments were performed in triplicate.

#### 2.3.6 Release Studies

The release studies were carried out by exposing the microcapsules to aqueous media.^1^ First, 50 mg of microcapsule slurry was placed inside a dialysis tube (length ∼2 cm, inner wet diameter ∼21.3 mm, uptake capacity ∼3.5 mL/cm, molecular weight cutoff 14 kDa, D004, BioDesign Dialysis Tubing, BioDesign Inc., NY, US). The tube was filled with 2 mL of ultrapure water. The outlets of each tube were secured with tight-grip lab pegs. Each tube was submerged in 50 mL of ultrapure water and stirred magnetically, with the proviso that the maximum possible concentration of HS from the microcapsules (*c*_max_) must be below the solubility threshold of HS in water (*c*_s_ ∼ 20 mg/L ≥ *c*_HS,max_ at 25 °C). Aliquots (1 mL) were sampled regularly over 20 days. Following each withdrawal, the same volume (1 mL) of fresh ultrapure water was replaced promptly into the receptor medium to maintain the sink condition. UV–vis spectra were recorded at 306 nm based on the relevant standard calibration curve (*R*^2^ ≥ 0.98), namely, HS-in-ultrapure deionized water. A control was performed using free oil (HS ∼ 3 mg, approximately corresponding to the active load of 50 mg of microcapsule slurry) within the dialysis tube to confirm that the oil could be entirely recovered on the outer phase. The moisture content of the microcapsule slurry (∼76%) was quantified using a rapid moisture analyzer (MA37, Sartorius, Göttingen, Germany, EU) via a gentle heating-drying method (103 °C) until no further mass change was detected.

#### 2.3.7 Characterization of the Mechanical Properties of Microcapsules Using Micromanipulation

The mechanical properties of microcapsules were assessed via a micromanipulation technique.^27,30^ Thirty microcapsules from each sample were individually compressed to achieve statistically representative results.^6,10^ The specimens were prepared by placing two droplets of microcapsule suspension onto a precut glass substrate (∼250 mm^2^) and allowing them to air-dry. The glass substrate was mounted onto the sampling stage perpendicular to a glass probe with a flat end of ∼90 μm in width. The probe was attached to the required transducer (model 405A 405017, maximum force scale 10 mN, Sensitivity 0.938 mN V^–1^, Aurora Scientific Inc., Canada). The force transducer was affixed to a three-dimensional fine micromanipulator operated by a servomotor (Parker Compumotor, USA) capable of controlling the vertical direction, moving distance, and compression speed. Each microcapsule was compressed at a speed of 2.0 μm s^–1^. The compression of microcapsules was monitored in real time by a side-view high-speed camera (charge-coupled device 4912-5010/000, Cohu, Poway, CA, US). The response generated from their compression resulted in a voltage-displacement data set, which was filed and further processed. Prior to testing, the compliance of the system was verified thrice, and the resulting mean value was used to calculate the actual displacement of the force probe.

## 3 Results and Discussion

### 3.1 Surface Modification of SiO_2_ NPs

**Figure 2:**
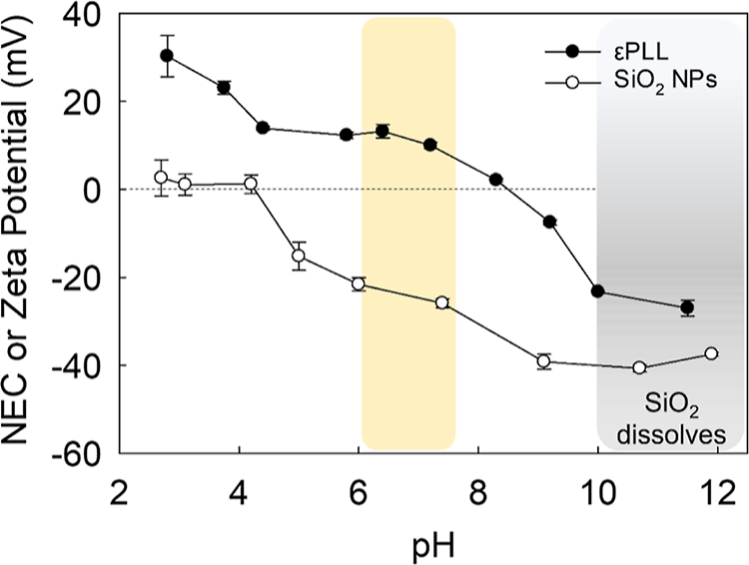
NEC of εPLL (soluble biopolymer) and zeta potential of SiO_2_ NPs (solid suspension) at different pH values. The vertical yellow strip represents the pH region (pH ∼ 6–7.5) at which the strength of electrostatic interaction between εPLL and SiO_2_ NPs is maximized.

The zeta potential of hydrophilic fumed nonmodified SiO_2_ NPs, as a function of pH, is displayed in Figure 2. At a low acidic pH around 2–4, the SiO_2_ NPs exhibited a near-zero surface charge, indicating proximity to the isoelectric region. At such low pH, both negative and positive charges generated by the silanol functional groups (Si–O–H) are equalized on the surface of the silica NP.^31^ This led to minimized repulsive forces between NPs and increased likelihood of aggregation. As the pH was increased above 4, the zeta potential became more negative, signifying an increase in negative charges at the particle surface. The greatest negative zeta potential was measured at highly alkaline pH of about pH 9 due to the complete deprotonation of silanol groups (SiO^–^).^31^ Beyond pH 10, it is noted that silica is prone to dissolving due to the potential reactions with hydroxide ions (OH^–^), forming soluble silicates.^32^ Moreover, silanol groups may undergo base-catalyzed reactions possibly leading to their decondensation and complete dissolution of silica in the presence of alkali hydroxides.^33,34^

In Figure 2, the NEC of εPLL is also shown. At the lowest pH values (pH ∼ 2–4), εPLL exhibited a highly positive charge. εPLL is a polycationic homopolypeptide periodically decorated with amine groups along its backbone. These groups become easily protonated at low pH levels. As a result, εPLL is expected to extend in a nonhelical conformation due to the electrostatic repulsion between its positive charges, leading to a completely soluble structure.^35^ The p*K*_a_ of εPLL was confirmed to be around pH 9, which allowed for successful electrostatic interactions with silica at a pH approximately between 6 and 7.5. Indeed, the surface modification was performed at pH 7, where the strength of electrostatic interaction between the two species is maximized.^10^

**Figure 3:**
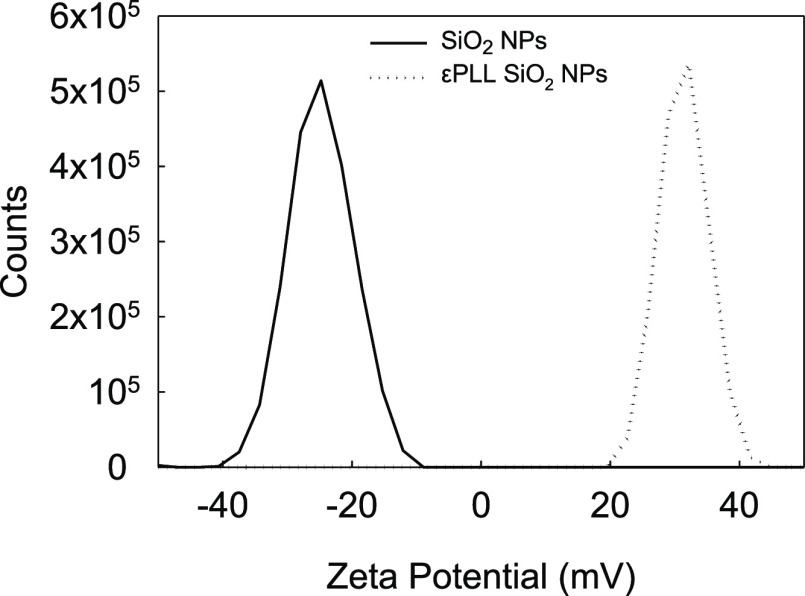
Zeta potential distribution of fumed SiO_2_ NPs before (—) and after surface modification with εPLL (···) at pH 7.

The zeta potential distribution of SiO_2_ NPs before and after surface modification with εPLL is depicted in Figure 3. Following the addition of εPLL, the surface charge of SiO_2_ NPs shifted from −24.4 ± 0.5 to 31.4 ± 0.1 mV, indicating the successful anchoring of εPLL to the SiO_2_ resulting in a net positive charge. Similar observations have been made by de la Torre et al.^36^ The authors customized mesoporous SiO_2_ NPs by capping εPLL onto their surfaces to facilitate the nanoencapsulation of pharmacological actives. In addition, the surface modification of SiO_2_ NPs with εPLL may have also played a key role in facilitating the subsequent condensation of inorganic materials like silica.^37^

### 3.2 Surfactant-Assisted Pickering Emulsion

**Figure 4:**
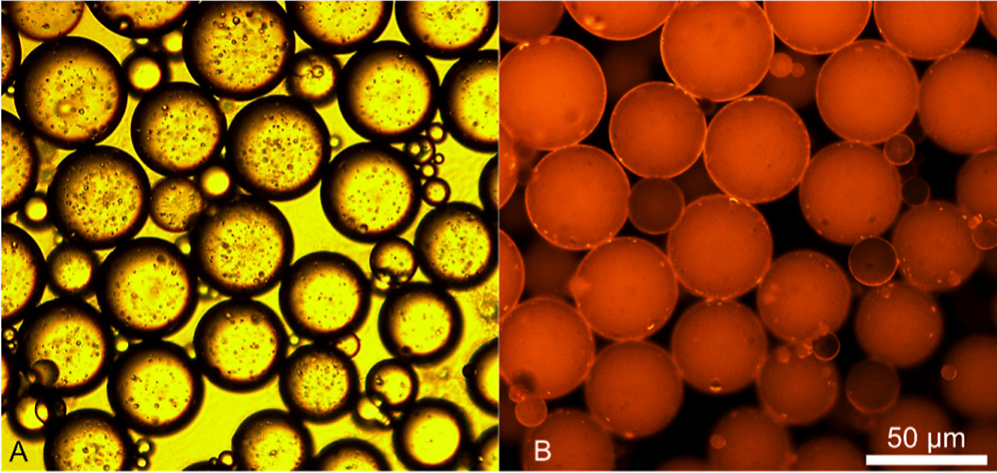
Stability of emulsified droplets prepared using SDS and εPLL-modified SiO_2_ NPs, affording both electrostatic and steric stabilization, under bright-field (A) and fluorescent light (B).

In Figure 4, o/w emulsion droplets are shown. These were prepared using SDS and εPLL-modified SiO_2_ NPs to possibly afford both electrostatic and steric stabilization. Under bright-field light (Figure 4A), the droplets appeared well dispersed. Despite being in close contact with each other, there was no evidence of droplet–droplet bridging, suggesting successful control over coalescence. The droplets presented in Figure 4A yielded a number-based mean diameter of 39.0 ± 1.2 μm, determined via ImageJ analysis. This was found to be unchanged after more than 1.5 h (Supporting Information, S1). This indicates that the droplets were kinetically stable if their encapsulation can be completed within this time frame. Interestingly, SiO_2_ NPs and SDS might have acted synergistically towards hindering droplet breakup and coalescence. Such a synergistic effect has been documented in literature, especially at concentrations below or equal to the cmc, yielding reduced interfacial tension values compared to solutions containing SDS alone.^38^ Indeed, emulsions concurrently decorated with SDS and SiO_2_ NPs, acting as a surfactant and cosurfactant, lead to armors on the droplet interface, increasing the emulsion stability.^39^ Tiarks et al.^40^ demonstrated that SDS and silica NPs acted in a synergistic fashion, serving as a surfactant and cosurfactant. Importantly, they elucidated that silica NPs alone were incapable of effectively stabilizing the emulsion at pH 3.

Besides, the surfaces of the droplets were predominantly marked by the presence of minuscule bubbles, likely stemming from the entrapment of air during the homogenization process. The SiO_2_ NPs capped with εPLL and featuring a net positive charge, likely played a role in regulating both electrostatic and electrosteric interactions at the interface of the oil/water droplets. This might have led to an attraction between the NPs, potentially culminating in the formation of a densely entangled network propelled by the electrostatic nature of SDS.

Within Pickering emulsions, fine colloidal particles, such as silica NPs, may not ensure stability if the surface of the emulsion droplets is patchily covered. Thus, an uneven coverage may result in droplet–droplet coalescence.^41^ Pickering NPs should not only adsorb promptly at the interface, but the o/w interface must also be sufficiently covered to generate an effective barrier. This would prevent the droplets from coalescing when in close proximity (Figure 4A,B). Therefore, it is essential to have a mono- or multilayer of charged, non-aggregated NPs, as reported for many systems, including sunflower oil or diisopropyl adipate with SiO_2_ NPs.^41,42^

The extent of interfacial coverage is conditional upon various factors, encompassing the electrostatic interplay between the solid and liquid species, the shape and size of the Pickering particles, as well as the processing conditions (pH, temperature, and ionic strength).^42,43^ Particles with a moderate charge (31.4 ± 0.1 mV) tend to arrange themselves in a tighter packing when compared to highly charged particles (>60 mV) that possess a longer range of electrostatic interactions.^44,45^ Thus, particles with a shorter range electrostatic interaction may form a denser pseudocontinuous monolayer at the droplet interface.^41^ Despite the infinitesimal distance between the oil droplets (Figure 4), there appeared to be no evident NP jamming and/or bridging between the interfaces of adjacent droplets, likely suggesting that the interdroplet liquid film may have been stabilized as well.^46^

The dark outlines observed on the droplets, as seen in Figure 4A, may be due to the εPLL-capped NPs possibly arranging into a spatial network, following the interfacial deployment of SDS. However, it is also plausible that the dark “rings” might be due to the effect of the fixed-position light source on the imaged droplets of similar size. Under fluorescence, a crimson signal was detected, indicating the presence of Nile Red (Figure 4B). Due to the sulfate functional groups, SDS molecules exhibit a consistently negative charge across a wide pH spectrum, with a NEC ranging between −38.2 ± 1.2 mV (pH 2–4) and −49.3 ± 2.3 mV (pH 6–10), as also reported by Loosli and Stoll.^47^ It is therefore acceptable to posit that the stability of the emulsion stems from the coherent NP layer and the surfactant film enveloping the droplets. These possibly acted as a combined mechanical-steric barrier and electrostatic shield against coalescence, yielding “armored” droplets.^48^

### 3.3 Primary Microcapsules

**Figure 5:**
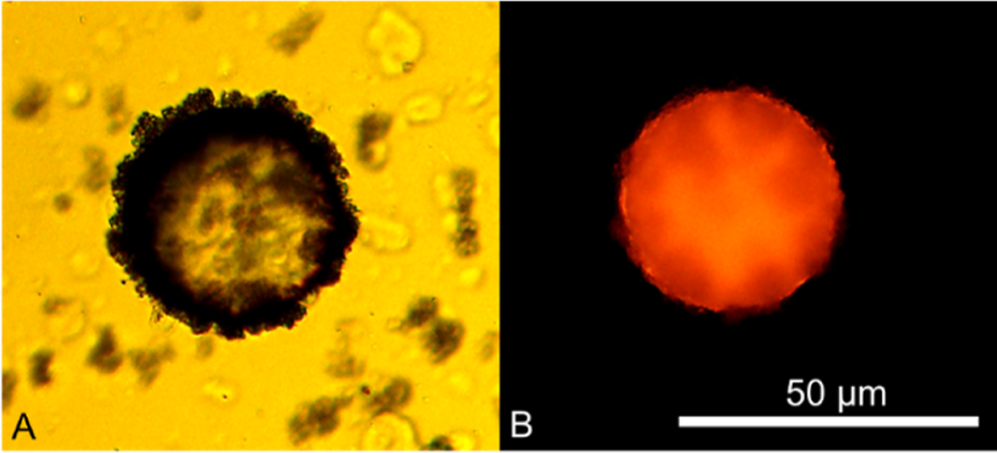
Optical (A) and fluorescent (B) microphotographs of a primary microcapsule formed by the electrostatic interaction between fCh and SDS.

A primary shell around the HS droplets was obtained upon application of fCh. The process was driven electrostatically, leveraging the positive charges carried by fCh at pH 2 against the residual negative charges administered by SDS (−38.2 ± 1.2 mV) and εPLL-SiO_2_ NPs (25.9 ± 1.9 mV).^10^ At such pH, fCh is completely solubilized, with protonated glucosamine groups (R-NH_3_^+^) leading to a fully extended conformation.^14^Figure 5 illustrates a typical primary microcapsule with a relatively spherical morphology when dispersed in water. Under bright-field light, its surface appeared to be moderately rough, without any evident cracks or wrinkles (Figure 5A). However, some fibrillar-like indentations at its peripheries were observed, yielding a discontinuous circular outline. The peripheral deformation of the microcapsule morphology may be attributed to the hydrophilic nature of fCh, which may form a hydrogel-based shell, as also reported by Omer et al.^49^ The lack of ideal sphericity, smoothness, and structural invaginations associated with chitosan structures was also documented in other works.^50^ However, it cannot be precluded that the presence of (poly)electrolytes, and the subsequent electrostatic interactions between positively and negatively charged groups, might have also triggered a local rearrangement of the NPs at the interface.^48^ This could potentially result in a NP multilayer via stacking or via heterogeneous aggregation at the interface. The biopolymerization of waterborne oligomeric chitosan radicals around armored Pickering emulsion droplets may have been aided by the presence of the SiO_2_ NPs, acting as interfacial mediators.^22^ This is possibly followed by the interfacial homocoagulation between the oligomeric chitosan radicals (biopolymerization) and/or heterocoagulation between SiO_2_ and polymerized chitosan.

Thongngam and McClements demonstrated that SDS can bind strongly to chitosan via a highly exothermic interaction, as investigated by isothermal titration calorimetry analysis. Consequently, this led to a slight increase in turbidity, indicating that the formed complex was likely insoluble.^51^

Jiang et al.^52^ reported that SDS binds to chitosan through interactions between alkyl-sulfate anions (R-SO_4_^–^) and amino cations (R-NH_3_^+^), resulting in an ionically cross-linked structure. This suggests that chitosan likely engages electrostatically with SDS, as well as its micelles, to form a networked hydrogel structure.^52^ Furthermore, SDS may also have been beneficial to attenuate the hydrogen–hydrogen interactions within chitosan, potentially rendering the biopolymer more suitable for binding to the oppositely charged polyelectrolytes. Fluorescence-assisted topographical analysis of the primary microcapsule revealed a denser and more compact structure, characterized by a smoother contour. The periphery of the microcapsule emitted a dimmed shade of red and appeared encircled by a thin film. When compared with Figure 5A, the outline appeared more homogeneous with no surface invaginations detectable under fluorescence. This suggests that aggregations/packings of inorganic material (i.e., SiO_2_ NPs), which are expected to be naturally nonfluorescent, were likely present.^13^

### 3.4 Dual-Shell Microcapsules

**Figure 6:**
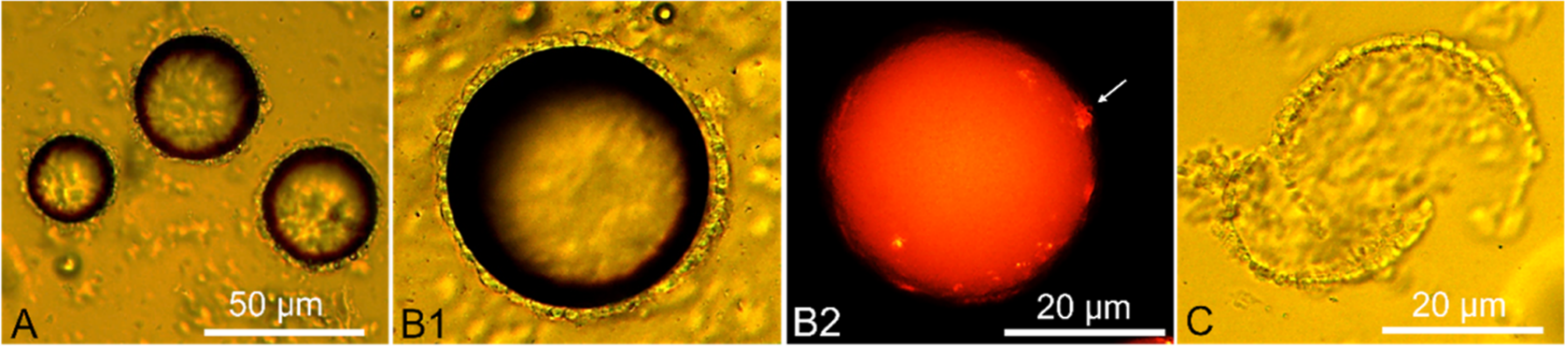
Overview of dual-shell composite microcapsules with the inorganic (SiO_2_) shell formed by external mineralization (A); close-up of a dual shell microcapsule with the inorganic (SiO_2_) crown detectable under bright-field light (B1) and undetectable under fluorescence (B2); and example of a detached SiO_2_ shell from an incomplete microcapsule (C). The white arrow in B2 highlights the presence of localized surface protuberances on the polymeric layer.

Dual-shell microcapsules were formed through the interfacial mineralization of SiO_2_. As shown in Figure 6A, the resulting microcapsules were spherical with an apparent core–shell configuration. An inorganic crown was formed cohesively around the primary microcapsules. Under microscopy, the inorganic crown appeared translucent (Figure 6B1), which is concordant with the nature of sodium silicate-based silica (water glass).^53^ When compared to the single-shell microcapsules (Figure 5), the dual-shell microcapsules appeared relatively compact, possibly due to the presence of self-assembled SiO_2_ crystals forming a hierarchical shell scaffolding. Unsurprisingly, the inorganic crystal-made crown was not detectable under fluorescence, as mesoporous silica in its mineralized solid form is unlikely to bind or solubilize dye molecules. In contrast, the diffusion of free silicate-bound dye molecules (e.g., Nile Red) in the presence of liquid-like silicate oligomers has been reported.^54^

Figure 6B2 displays a relatively smooth surface, which is ascribable to fCh being contained within the silica crown. This fCh coating appeared very different to that of single-shell microcapsules (Figure 5A). It is conceivable that the inorganic crown may play an important role in molding/reshaping highly pliable hydrogel structures, such as fCh. Therefore, the interactions with the newly formed silica layer likely reduces the degree of freedom of the polymer film, making it more compact. However, several surface protuberances (see white arrow) were also visible, possibly indicating an inhomogeneous distribution of the silica NPs embedded within the hydrogel matrix. The precipitation of mineralized silica around fCh was indeed facilitated by the presence of silica NPs that acted as nucleation kernels for the growth of silica crystals into a compact shell. Similar seed-mediated growth mechanisms have been previously observed, with particular emphasis on noble metal NPs in the presence of a reducing agent.^55^

In our study, mineralized silica crystals were attained via the acidic hydrolysis of sodium silicate.^56^ When gradually inoculated into the primary microcapsule suspension at pH 2, sodium silicate (Na_2_SiO_3_) may first react with water in the presence of an acid (HCl_aq_ → H^+^ + Cl^–^) to form an intermediate, namely, metasilicic acid, and a sodium salt (Na_2_SiO_3_ + 2HCl → H_2_SiO_3_ + 2NaCl). This is followed by its hydrolytic condensation to yield silica as the final product (H_2_SiO_3_ → SiO_2_ + H_2_O). The silica crystals combined into a three-dimensional spherical shell are visible in Figure 6C, where an incomplete microcapsule (without any oil core) is presented. The shell appeared fractured into two pieces, likely due to the mechanical application of a glass slip onto the specimen during observation. Notwithstanding, both halves of the shell were optically clear, and displayed continuous curvatures developing across distinct spatial planes, allowing for the identification of the prior oil-hosting pocket.

### 3.5 SEM Analysis

**Figure 7:**
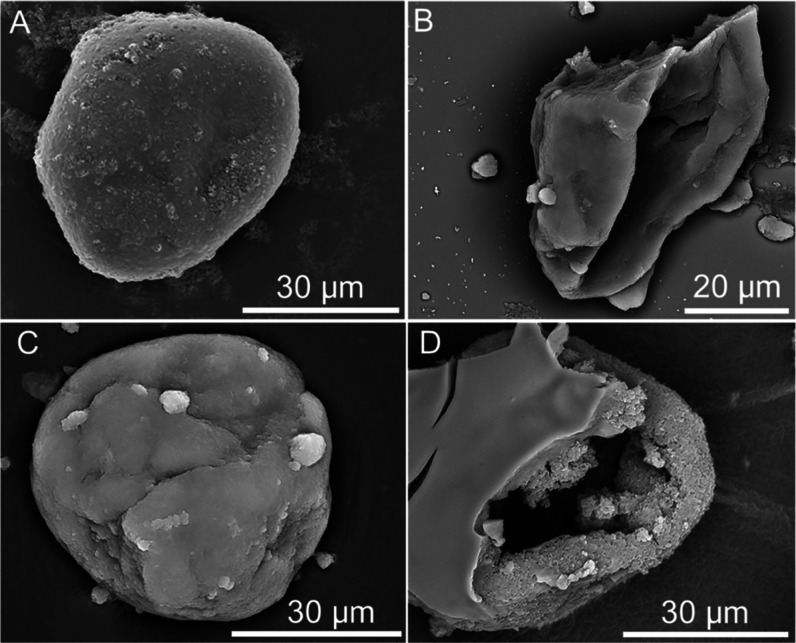
SEM micrographs of (A) complete and (B) incomplete single-shell (fCh) microcapsule and of (C) complete and (D) (incomplete) dual-shell (fCh-SiO_2_) dual-shell microcapsule.

The SEM micrograph of a single-shell (fCh) microcapsule is presented in Figure 7A. Although relatively spherical, the periphery of the microcapsules was found not to be completely circular. This seemed not fully consistent with the images from optical microscopy (Figure 5A). Upon drying and under vacuum conditions (<10^–3^ Pa), a morphological deformation of single-shell microcapsules is plausible due to the hydrophilic nature of the fCh-based hydrogel network. Therefore, water loss may lead to structural shrinkage/contraction, as also reported elsewhere.^49^ Notwithstanding, the structure was relatively compact, without any visible surface rippling. However, there appeared to be multiple surface granules, potentially originating from the aggregation or stacking of SiO_2_ NPs. As mentioned above, this may be attributed to the electrostatic rearrangement between the polyelectrolytes and/or some physical entanglement within the fCh network during encapsulation.

Figure 7B illustrates an incomplete single-shell microcapsule. An internal pocket was discernible, confirming the core–shell structure of such microcapsules. This finding aligns with the observations from Figure 5A,B. Both inner and outer surfaces were visually smooth and dense. The corresponding EDX analysis detected a high concentration of atomic carbon (∼63%) on the external side presumably due to the prevalence of chitosan. Elemental silicon was also detected (∼36%), possibly suggesting that the fumed NPs may have partly embedded within the fCh matrix. However, because the detecting depth of X-rays is within the range of a few hundred nanometers to 1–2 μm,^57^ it cannot be excluded that the signal detected through the shell might originate from the fumed silica NPs interfacially deployed at the inner surface (Supporting Information, S1A). In contrast, the internal side was dominated by atomic silicon (∼79%), with carbon accounting for only ∼9% of the atomic composition (Supporting Information, S1B). This result was not surprising because Pickering NPs were anticipated to adsorb at the o/w interface, which corresponds to the periphery of the empty pocket observed in Figure 7B.

A typical dual-shell microcapsule is depicted in Figure 7C, which possessed a relatively spherical morphology with a reasonably smooth surface. However, a topographical analysis revealed a few rougher areas, denoted by the white arrow. This inhomogeneity may be due to the presence of those silica crystals that were incapable of merging into a continuous inorganic network. Alternatively, we postulated that a cohort of crystals might not have been able to ripen uniformly, leading to partially accreted crystals which arranged into a coarser surface.^20^ Several large surface beads (∼1–4 μm) were also observed. These may have been due to the “overaccretion” of certain crystals via a NP-mediated self-assembly. However, these beads were spherically shaped, which suggests that they may have attached to the microcapsule surface at a later stage during the secondary encapsulation process. This is possibly a consequence of the repositioning/realignment of the excess silicate material.^1^

Figure 7D shows part of an incomplete dual-shell microcapsule. A large, deep pocket was observed, providing further evidence for the core–shell nature of these microcapsules. Notably, the texture of the inner and outer shells appeared different. The outermost surface was fairly smooth, which is concordant with the observation from Figure 7C. EDX analysis revealed that the surface was mainly composed of atomic carbon (∼74%), which is likely ascribable to chitosan (Supporting Information, S2C). Several irregular surface depressions/ruptures were also visible, possibly induced by the vacuum conditions within the SEM chamber. Alternatively, these contractions might have stemmed from the spontaneous partial deflation of the microcapsule structure upon the release of the core as a result of structural damage caused by the vacuum. As with Figure 7C, multiple discrete irregularly shaped fragments were also identified at the surface of the inner coating. Based on EDX analysis, these are predominantly carbon-based (∼67%), indicating that they likely originated from the debris of the chitosan coating (Supporting Information, S2D). In contrast, the inner surface of the microcapsule (pocket) appeared inherently porous, which is likely related to the self-assembly of silica crystals. Indeed, it was determined that the inner lining primarily consisted of atomic silicon (∼64%) and carbon (∼35%). Similarly, the outer surface of the inner coating comprised silicon (∼48%) and carbon (∼30%), probably attributable to inorganic silica crystals and εPLL-capped SiO_2_ NPs (Supporting Information, S2E,F). This leads to the hypothesis that silicates may have migrated inward to form the inorganic coating, possibly due to the presence of SiO_2_ NPs adsorbed at the oil/water interface. Thus, it is conceivable that sodium silicate might have permeated through the primary fCh-hydrogel layer to meet the nucleation seeds (i.e., SiO_2_ NPs) at the interface. This interaction, in turn, may have instigated the accretion of crystals from within. In a similar vein, Long et al. engineered double-coated microcapsules with calcium carbonate (CaCO_3_) and melamine–formaldehyde (MF).^20^ The authors illustrated that the MF layer was evenly smooth, while the CaCO_3_ appeared coarse. This was attributable to the ripened CaCO_3_ crystals assembling into a discontinuous and inherently porous framework. Despite the application of MF subsequent to the formation of the CaCO_3_ shell, it evidently migrated inward through the pores to form the synthetic coating at the oil/water interface.

### 3.6 Particle Size Distribution

**Figure 8:**
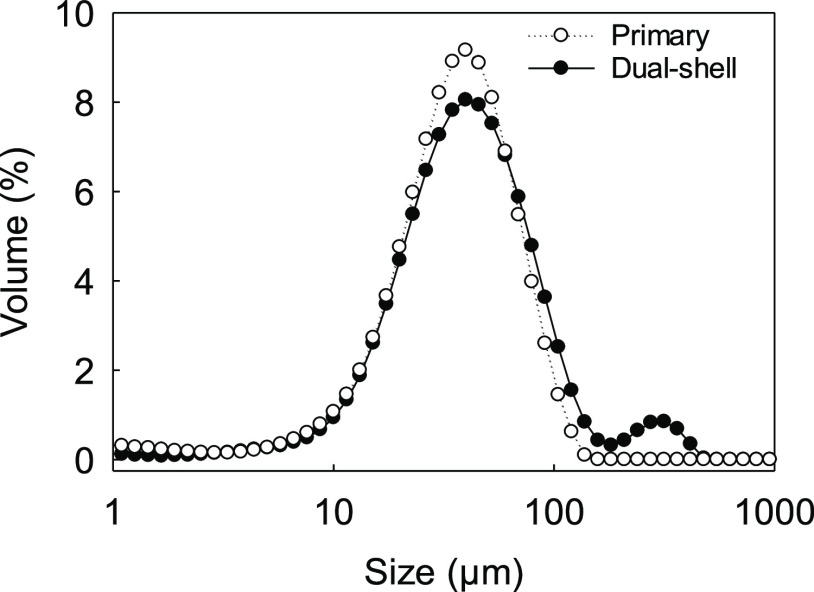
Particle size distribution of primary and dual-shell microcapsules obtained via laser diffraction.

The Sauter diameter (*D*_[32]_) of single-shell microcapsules was 42.3 ± 0.4 μm, whereas dual-shell microcapsules exhibited a slightly larger diameter (*D*_[32]_ = 51.4 ± 0.4 μm). This variation may be attributed to the formation of SiO_2_ crystals, which led *D*_[32]_ to increase by ∼18% on average. The particle size distribution (PSD) of single-shell microcapsules was found to be unimodal and relatively narrow (Figure 8). The PSD of dual-shell microcapsules did not change significantly when compared to that of the primary microcapsules. However, a minor secondary peak with lower intensity was also observed. This peak may be attributed to the presence of inorganic aggregates in bulk, possibly due to an excess silicate material. This is reflected in the significantly increased *D*_[32]_ of dual-shell microcapsules, which indeed accounts for the presence of free aggregates in-bulk, other than the addition of a secondary silica layer on the microcapsules.

### 3.7 Mechanical Properties

**Figure 9:**
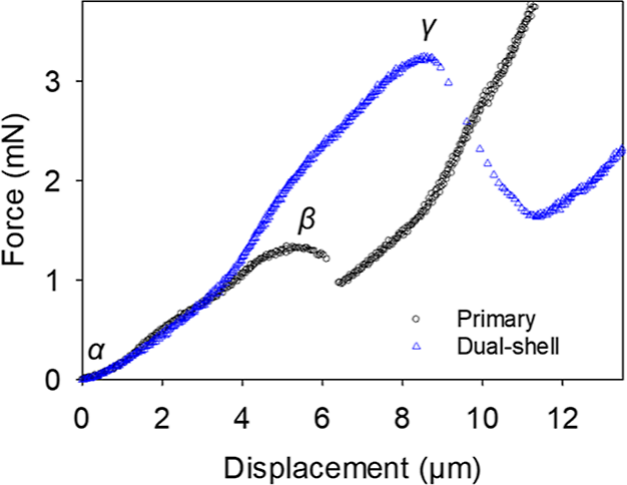
Example of a force–displacement curve of primary (○) and dual-shell microcapsules (Δ) of similar size (∼35 μm). The symbol α represents the onset of compression, whereas β and γ are the recorded rupture points of primary and dual-shell microcapsules, respectively.

Figure 9 shows typical force–displacement curves for single- and dual-shell microcapsules under compression. The segments α–β and α–γ are monotonically positive and correspond to the progressive compression of individual microcapsules of a similar size (∼35 μm). At point β and γ, a drop in the applied force was observed, indicative of microcapsule rupture by compression. As shown, the force–displacement response generated by the dual-shell microcapsule was significantly different from that of the primary microcapsule. When compared to each other, the rupture force and resulting displacement at rupture of the dual-shell microcapsule were both significantly greater. This is possibly due to the presence of a composite shell, with a silica coating. Therefore, a deeper penetration of the probe into the shell, and consequently a larger force, are required for the dual-shell microcapsule to rupture. Moreover, the sudden drop in force measured at rupture was also more intense, suggesting a significant structural difference between single- and dual-shell microcapsules. This is due to the nature of the inorganic crystals present in the dual-shell capsules that possess a crystalline lattice and are inherently robust. However, they typically fracture catastrophically in a shattering mode under sufficient compression. This was caused by cleavage, cracking and splitting, triggered by external loading (i.e., the probe).^58^ By contrast, fungal chitosan is a biopolymer which exhibits rubber-like behavior.^6^ This yielded a residual force (β) in the single-shell microcapsule, resulting in a lower drop in measured force, and possibly a more elastic response under compression, as also observed in previous works.^10^ It is noted in Figure 9 that the initial slope of both the curves appears to overlap. However, this is not a generic observation as Figure 9 only displays an example of a force–displacement curve for one primary and one dual-shell microcapsule with similar sizes (∼35 μm). The two curves can significantly differ from each other because of onset of compression depending on individual microcapsules chosen (Supporting Information, S3).

**Figure 10:**
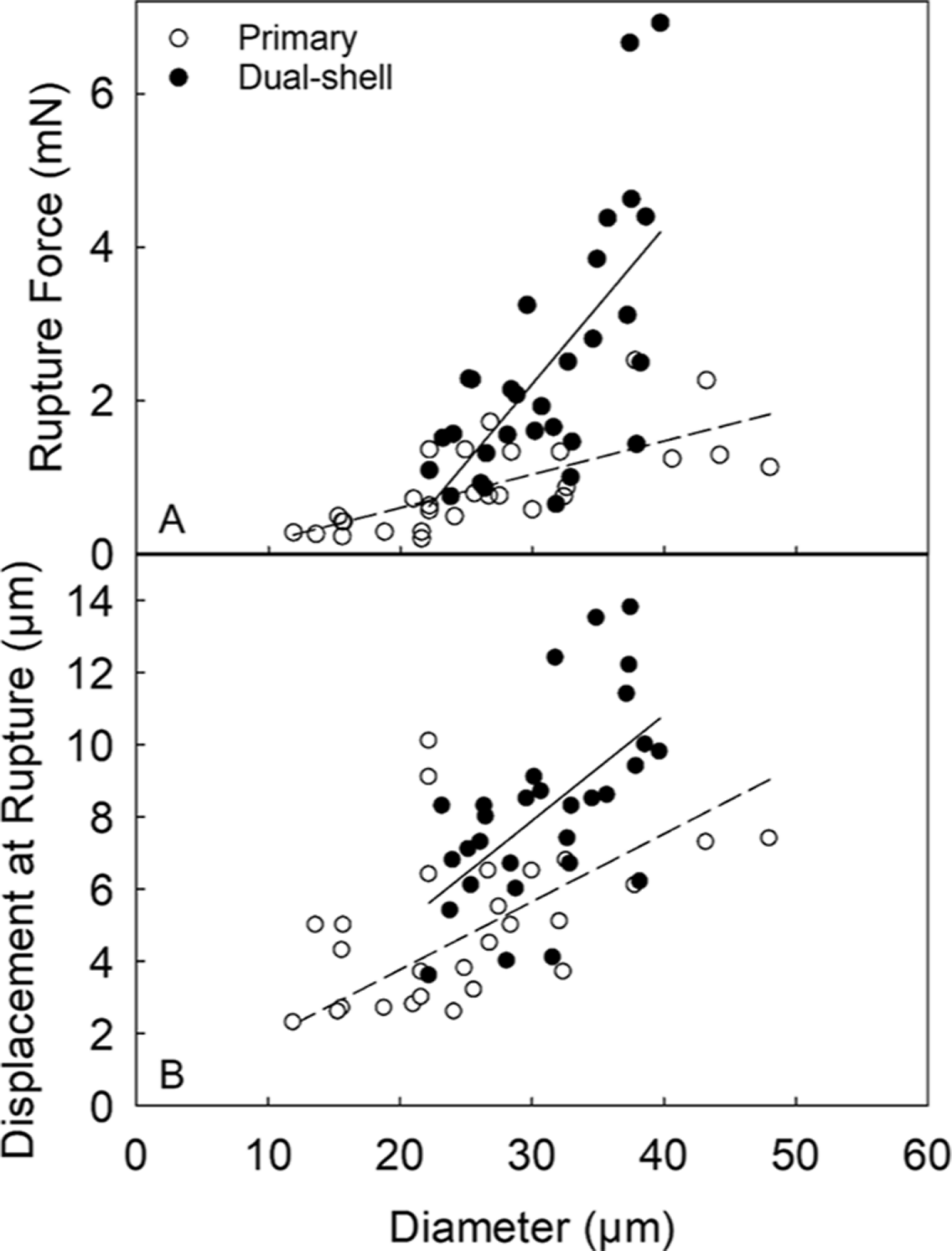
Comparison between the key diameter-dependent mechanical property parameters of primary and dual-shell microcapsules: rupture force (A) and displacement at rupture (B) vs capsule diameter. The dash (--) and solid (—) linear regressions represent the trend only, and correspond to primary and dual-shell microcapsules, respectively.

In Figure 10, the required force for rupture and associated displacement of 30 microcapsules from each sample as a function of the microcapsule diameter are displayed. The rupture force of both single- and dual-shell microcapsules was found to increase with diameter. When compared to each other, the resulting regression lines were significantly different (Figure 10A). The line resulting from dual-shell microcapsules was much steeper than that from single-shell microcapsules. This indicated that the dual-shell microcapsules generated a greater mechanical response. Because no statistically significant difference between the number-based diameter of the selected single- (26.6 ± 1.8 μm) and dual-shell microcapsules (31.1 ± 1.0 μm) arose, their mean rupture force values could be directly compared. Dual-shell microcapsules were stronger (2.44 ± 0.29 mN) approximately by 150% than the single-shell ones (0.99 ± 0.16 mN), with 95% confidence. Under compression, dual-shell microcapsules yielded a mean nominal rupture stress of 3.0 ± 0.2 MPa, which is significantly higher than that of single shell ones (1.7 ± 0.1 MPa), as shown in Table 1. Figure 10B shows the relationship between displacement at rupture of microcapsules as a function of the diameter. For both types of microcapsules, displacement is proportional to capsule diameter on average, as also reported in previous studies.^1,59^ Interestingly, there seemed to be overlapping of some experimental data points between single- and dual-shell microcapsules across the investigated diameter range. However, there appeared to be a significant difference in the slope of the regression lines. Overall, the displacement of dual-shell microcapsules appeared to be vertically shifted toward higher values. This suggested that the presence of an additional coating actively contributed to forming more solid microcapsules with a thicker shell, which required a deeper indentation for rupture. This indicates that the inorganic SiO_2_ crystal coating effectively enhanced the mechanical properties of the microcapsules.

**Table 1:** Key Mechanical Property Parameters of Primary and Dual-Shell Microcapsules (Mean ± Standard Error)

	primary	dual shell
number-based diameter (μm)	26.6 ± 1.8	31.1 ± 1.0
rupture force (mN)	0.99 ± 0.16	2.44 ± 0.29
rupture tension (N m^–1^)	37.3 ± 3.9	78.6 ± 7.2
nominal rupture stress (MPa)	1.7 ± 0.2	3.0 ± 0.2
displacement at rupture (μm)	5.6 ± 0.5	8.2 ± 0.5
deformation at rupture (%)	21.4 ± 1.7	26.3 ± 1.2

### 3.8 Barrier Properties

**Figure 11:**
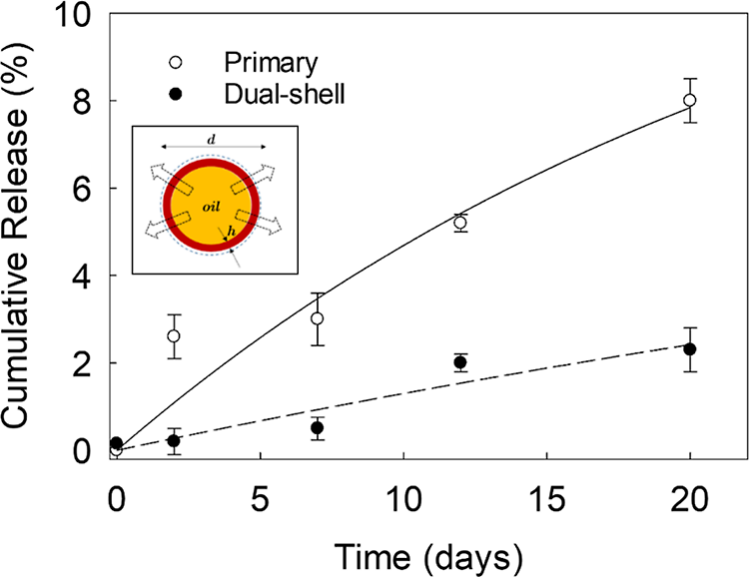
Release profile of HS through the microcapsule shells in water. The dashed line displays the trend of the profiles fitted to a nonlinear regression model i.e. *R*(*t*) = *a*(1 – e^–*t*/τ^), where *R*(*t*) [dimensionless] is the amount oil cumulatively released (operative range 0–1 or 0–100%) at time *t* [days], τ is the characteristic diffusion time [days], and *a* [dimensionless] is the pre-exponential factor associated with each single microcapsule, representing the amplitude of the diffusional process. The schematic inset represents the release mechanism of a microcapsule with a given diameter (*d*) and shell thickness (*h*).

An active ingredient may be released through the microcapsule shell conditionally based upon the nature of the shell and the active solubility in the receptor medium and within the shell itself. Here, both single- (payload 35.5 ± 4.5% and EE 47.8 ± 6.2%) and dual-shell microcapsules (payload 27.6 ± 1.3% and EE 52.1 ± 9.9%) were assayed for their oil release in fresh neutral-pH aqueous environments (Figure 11). After 24 h, ∼3.5% of HS was leaked out from single-shell microcapsules, while ∼1.5% was leaked from dual-shell microcapsules. Interestingly, these findings suggested that the presence of an additional silica coating provided the shell with an additional mass-transfer resistance, thereby slowing down the diffusion of oil through the shell itself. These values were not substantially different to those of microcapsules with a MF-shell, which typically release around 0.5% of the core oil after 24 h in water.^16^ After 20 days in water, it was found that only ∼2.5% was released from dual-shell microcapsules, whereas single-shell microcapsules cumulatively released ∼10%. In addition, when compared to both primary and dual-shell microcapsules, it is worth mentioning that mass transfer of free oil (HS) within the dialysis tubing (i.e., control) into the receptor medium would occur at a more rapid rate, resulting in over 40% leakage after 2.5 h (Supporting Information, S4). This release rate is significantly higher than the release of oil from microcapsules, despite an equivalent initial oil load. In other words, the mass-transfer resistance posed by the dialysis tubing is insignificant and can be ignored, signifying that the microcapsule shells were able to impart substantial resistance to the mass transfer of encapsulated oil into the receptor medium.

The corresponding characteristic diffusion times were determined by nonlinear regression (see the caption of Figure 11), yielding 22.4 ± 3.7 and 43.2 ± 4.1 days for the primary and dual-shell microcapsules, respectively, with 95% confidence. Interestingly, the characteristic diffusion time associated with dual-shell microcapsules appeared to be around 2-fold of that of the primary capsules. This suggested a slower diffusion rate of the oil (HS) through the dual shell into the aqueous receptor medium. It is plausible that the presence of an additional inorganic coating atop the fungal chitosan shell may be conducive to mitigating oil leakage. However, the analysis of two characteristic diffusion times did not reveal a dramatic difference between them, suggesting that the chitosan shell might predominantly act as the principal mass (oil)-transfer resistance. This is not surprising because silica crystals are known to form noncontinuous/porous structures, offering limited contribution to reducing leakage.^17^ In contrast, chitosan may form smooth and homogeneous structures that are more suitable for the retention of oil. It is worthwhile mentioning that some oils may also be solubilized within the biopolymeric network, therefore partially wetting the inner layers, although more research is required to validate this.^6^ These observations are reflected in the value of “*a*” in the equation to describe the diffusional process of the oil through the shell (see the caption of Figure 11). Specifically, the determined values of “*a*” were 12.9 ± 0.9 and 5.5 ± 0.7% for primary and dual shell microcapsules, respectively. It is worth noting that neither of these values approached 100%, underscoring the impaired capability of the oil to diffuse through these shells into the receptor medium. It is important to mention that the value associated with dual-shell microcapsules was approximately half that of the primary ones, indicating that the supplementary silica coating likely played a role in further decelerating the diffusional process. It is imperative to note that our findings are system specific and associated with HS as a model ester oil, which is broadly used in the cosmetic and fragrance industry due its stability and low volatility. The encapsulation of other actives, such as essential oils (EOs), may lead to slightly/significantly different performance properties, possibly due to the presence of terpenic molecules. Our previous studies showed that terpene-rich EOs, such as l-carvone^1^ and limonene,^13^ impacted the interfacial energy and, consequently, the stability of the emulsions. This resulted in distinct mechanical properties observed among microcapsules possessing identical shell chemistry but varying core materials.^14^ Overall, these results seem to confirm that the silica coating applied to HS-entrapping primary microcapsules contributed to achieving a longer-term sustained release, along with enhanced mechanical properties, which may be advantageous for capsules deployed in consumer products. As a step forward toward developing sustainable and fully consumer-friendly products, SDS should be replaced with non-irritant natural anionic surfactants, such as sodium lauroyl methyl isethionate and sodium cocoyl isethionate, which are derived from coconut and more suitable for skin, scalp, and other cosmetics applications.^60^

## 4 Conclusions

We have designed and implemented an economical and environmentally conscious methodology for fabricating composite microcapsules with a dual organic–inorganic shell for oil delivery. These capsules possess a dual shell made of vegetable chitosan and silica, featuring a core of model fragrance oil (HS). Individual microcapsules with a relatively spherical morphology were fabricated. Single-shell microcapsules possessed a Sauter diameter of 42.3 ± 0.4 μm, whereas dual-shell microcapsules exhibited a slightly larger Sauter diameter (51.4 ± 0.4 μm) on average. Under compression, the dual-shell microcapsules yielded a mean nominal rupture stress of 3.0 ± 0.2 MPa, which is significantly greater than that of the single-shell counterparts (1.7 ± 0.2 MPa), with 95% confidence. Quantitative evaluation of fragrance release from within the microcapsules to surrounding water was performed. After 20 days in neutral pH water, only ∼2.5% of HS was released from the dual-shell microcapsules, while the single-shell ones released around 10%. The characteristic diffusion times of single- and dual-shell microcapsules were 22.4 ± 3.7 and 43.2 ± 4.1 days, suggesting that the supplementary silica coating played a significant role in improving the barrier properties of the capsule. Overall, the dual shell not only enhanced the mechanical performance of microcapsules but also improved their barrier properties in comparison with the corresponding single-shell microcapsules, aligning with those of commercially available microcarriers based on synthetic polymers (e.g., MF). This renders these microcapsules as a potentially valuable delivery system of oil-based active ingredient for consumer products. Future work will be directed at (i) making more compact and homogeneous silica shell in order to further improve the mechanical and barrier properties of these microcapsules, and (ii) enhancing the formulation using fully consumer-friendly, biodegradable, and plant-sourced surfactants, such as sodium cocoyl isethionate, which is gentler on both the skin and scalp.

## Supporting Information Available

Analysis of the diameter of the emulsion droplets by ImageJ; EDX analysis of the outer and inner side of incomplete single-shell microcapsule as presented in Figure 7B; EDX analysis of outermost surface, outer fragments, inner pocket, and outer surface of an incomplete dual-shell microcapsule, as presented in Figure 7D; comparison of force–displacement curves for primary and dual-shell microcapsules of similar size (∼25 μm); and cumulative release of free oil (HS) within the dialysis tubing (i.e., control) into the receptor medium resulting in over 40% leakage after 2.5 h (PDF)
