# Supplementary material for: Eco-Friendly Fungal Chitosan-Silica Dual-Shell Microcapsules with Tailored Mechanical and Barrier Properties for Potential Consumer Product Applications
Source: ACS Omega. 2024 Jun 20;9(26):28385–96. doi: 10.1021/acsomega.4c02287 (PMC11223154; doi:10.1021/acsomega.4c02287)
Supplement: Supplementary file 1 — ao4c02287_si_001.pdf [file ao4c02287_si_001.pdf]

Eco-Friendly Fungal Chitosan-Silica Dual-Shell Microcapsules with Tailored Mechanical and Barrier Properties for Potential Consumer Product Applications

Daniele Baiocco[1], Mohammed Al-Sharabi[2], Benjamin T. Lobel[3], Olivier J. Cayre[3], Alexander F. Routh[2], Zhibing Zhang[1*]

[1]School of Chemical Engineering, University of Birmingham, Birmingham, B15 2TT, UK
[2]Department of Chemical Engineering and Biotechnology, University of Cambridge, Cambridge CB3 0AS, UK.
[3]School of Chemical and Process Engineering, University of Leeds, Leeds LS2 9JT, UK

*e-mail: z.zhang@bham.ac.uk

Supplementary Material

Contains:

Table S1 – Analysis of the diameter of the emulsion droplets by Image J.

Figure S1 – EDX analysis of the outer (A) and inner (B) side of incomplete single-shell microcapsule as presented in Figure 7B

Figure S2 – EDX analysis of (C) the outermost surface, (D) the outer fragments, (E) inner pocket, and (F) outer surface of an incomplete dual-shell microcapsule as presented in Figure 7D.

Figure S3 – Comparison of force-displacement curves for primary (A) and dual-shell microcapsules (B) of similar size (~25 μm).

Figure S4 – Cumulative release of free oil (HS) within the dialysis tubing (i.e. control) into the receptor medium resulting in over 40% leakage after 2.5 hours.

Table S1: Analysis of the number-based diameter (NBD) of the emulsion droplets by ImageJ.

**Count**	NBD at time zero (µm)	NBD at 90 min (µm)
1	47.1	33.2
2	46.2	31.7
3	38.4	28.1
4	42.4	29.3
5	42.3	48.3
6	39.8	41.6
7	40.7	50.4
8	40.9	43.5
9	42.9	46.1
10	44.0	44.5
11	38.9	43.1
12	44.2	42.7
13	40.7	43.8
14	42.4	44.8
15	42.0	44.7
16	38.3	41.5
17	39.2	41.2
18	35.9	41.7
19	35.4	40.7
20	32.5	38.8
21	36.0	35.1
22	30.6	37.0
23	40.5	35.8
24	37.7	33.2
25	16.5	34.8
**Mean**	**39.0**	**39.8**
**St. Deviation**	5.7	5.6
**St. Error**	**1.2**	**1.1**

Spectrum details

Fungal Chitosan (single layer) New project   Spectrum name A, B

Electron Image

Image Width: 106.5 µm

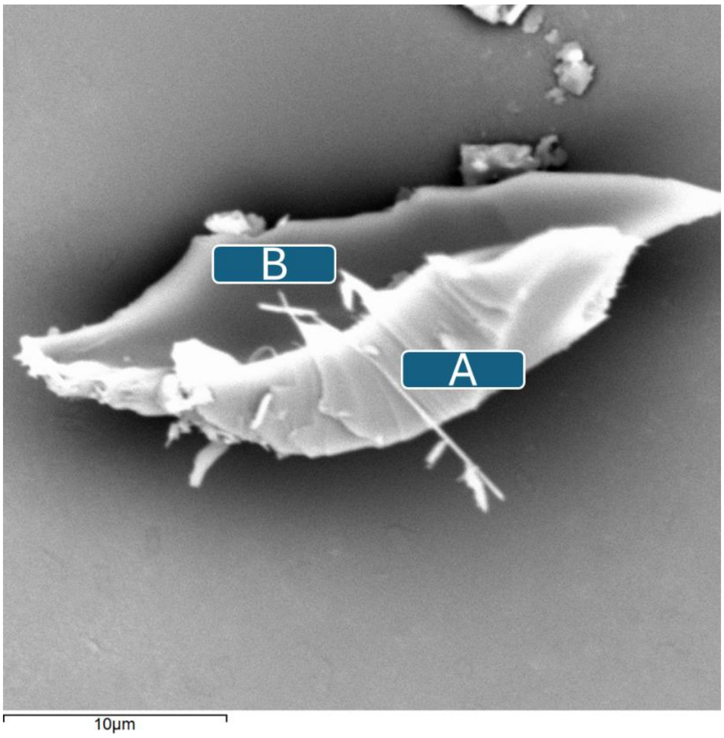

Acquisition conditions

Acquisition time (s) 20.0   Process time 5

Accelerating voltage (kV)   15.0

Summary results (A)

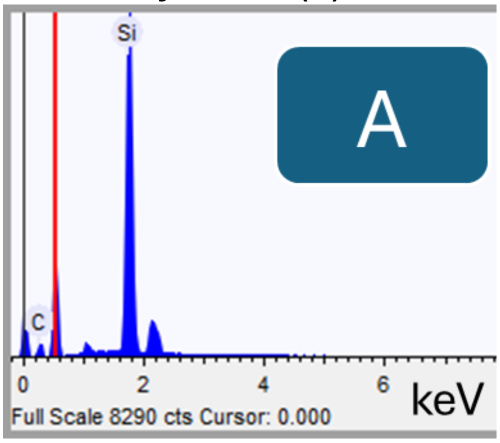

Element	Atomic %
Carbon	64.396
Silicon	35.604

Summary results (B)

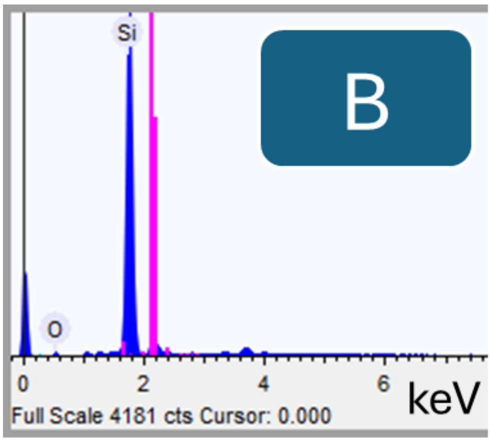

Element	Atomic %
Carbon	8.934
Oxygen	12.159
Silicon	78.907

Figure S1 – EDX analysis of the outer (A) and inner (B) side of incomplete single-shell microcapsule as presented in Figure 7B.

Spectrum details

Project New project Spectrum C

Electron Image

Image Width: 88.8 µm

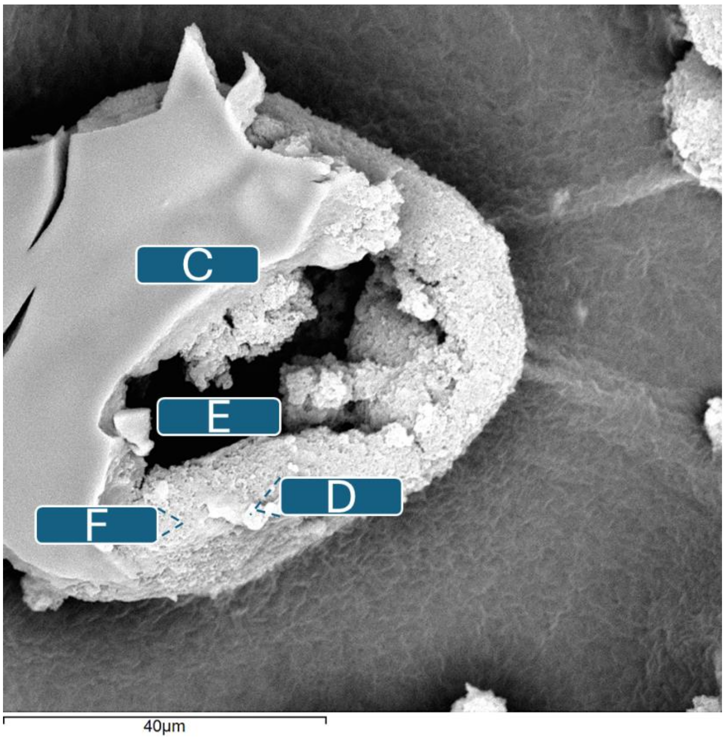

Acquisition conditions

Acquisition time (s) 20.0 Process time 5

Accelerating voltage (kV) 15.0

Summary results (C)

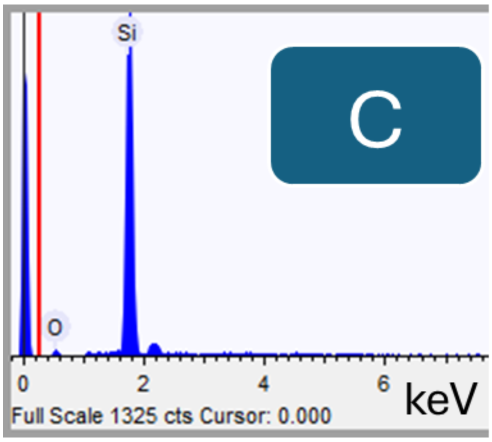

Element	Atomic %
Carbon	73.663
Oxygen	9.708
Silicon	16.629

Summary results (D)

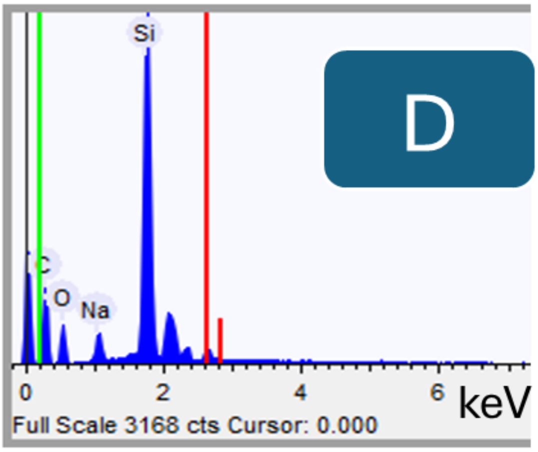

Element	Atomic %
Carbon	66.476
Oxygen	18.360
Silicon	13.554
Sodium	1.610

Summary results (E)

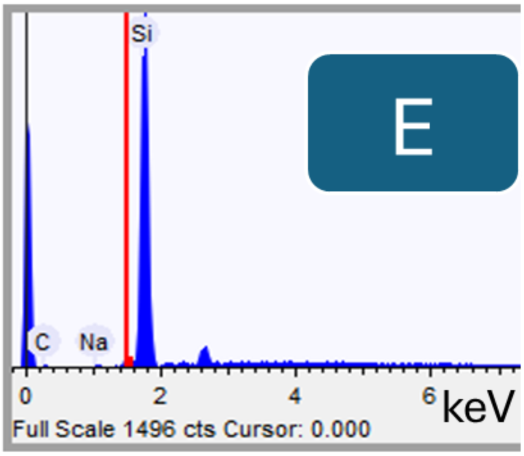

Element	Atomic %
Carbon	35.981
Sodium	0.647
Silicon	63.372

Summary results (F)

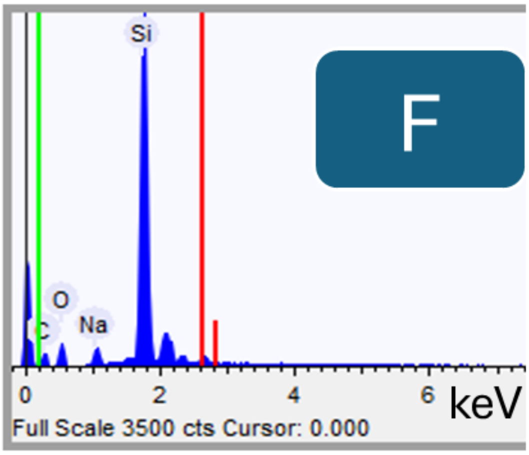

Element	Atomic %
Carbon	29.675
Oxygen	20.141
Sodium	2.504
Silicon	47.680

Figure S2 - EDX analysis of (C) the outermost surface, (D) the outer fragments, (E) inner pocket, and (F) outer surface of an incomplete dual-shell microcapsule as presented in Figure 7D.

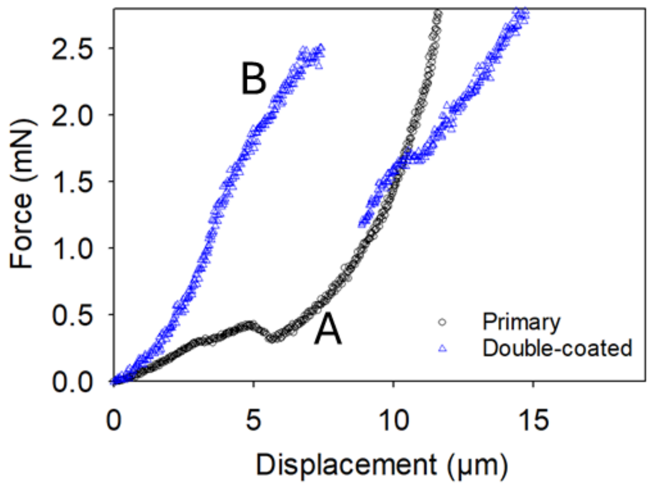

Figure S3 – Comparison of force-displacement curves for primary (A) and dual-shell microcapsules (B) of similar size (~ 25 μm).

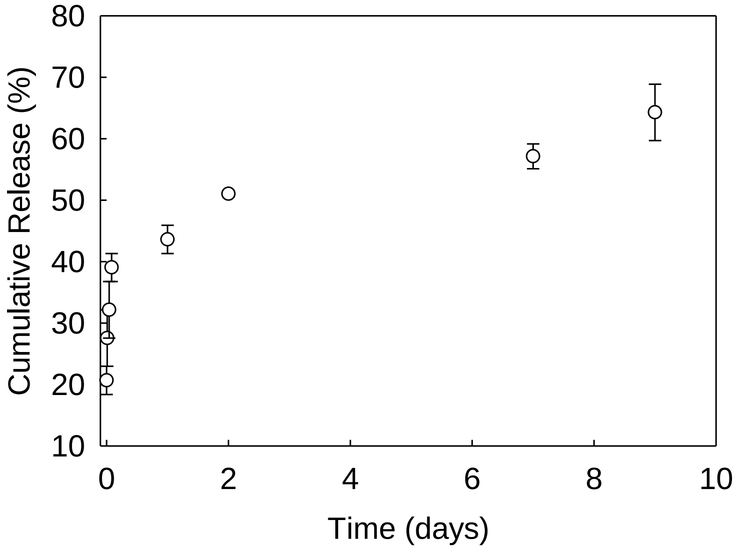

Figure S4 – Cumulative release of free oil (HS) within the dialysis tubing (i.e. control) into the receptor medium resulting in over 40% leakage after 2.5 hours.
